# MicroRNAs get to the heart of development

**DOI:** 10.7554/eLife.01710

**Published:** 2013-11-19

**Authors:** Ge Tao, James F Martin

**Affiliations:** 1 **Ge Tao** is in the Department of Molecular Physiology and Biophysics, Baylor College of Medicine, Houston, Texas, United Statesge.tao@bcm.edu; 2 **James F Martin** is in the Department of Molecular Physiology and Biophysics, and the Program in Developmental Biology, Baylor College of Medicine and the Texas Heart Institute, Houston, Texas, United Statesjfmartin@bcm.edu

**Keywords:** microRNA-1, cardiac, sarcomere, Telokin, genetics, smooth muscle gene expression, Mouse

## Abstract

A microRNA regulates the expression of a network of genes in the heart to ensure that progenitor cells develop into strongly contractile cardiac muscle.

**Related research article** Heidersbach A, Saxby C, Carver-Moore K, Huang Y, Ang YS, de Jong PJ, Ivey KN, Srivastava D. 2013. microRNA-1 regulates sarcomere formation and suppresses smooth muscle gene expression in the mammalian heart. *eLife* **2**:e01323. doi: 10.7554/eLife.01323

**Image** Mice that lack microRNA-1 show abnormal development of the heart (right) compared to wildtype animals (left). (RA and LA–right and left atrium; RV and LV–right and left ventricle)

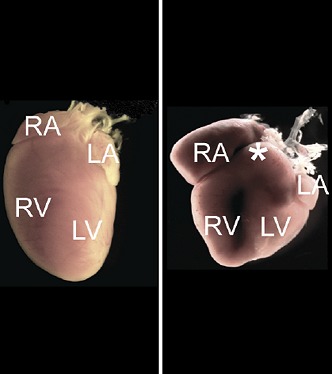

The heart is the first organ to form in mammalian embryos. In mice, the heart starts to pump blood shortly after embryonic day 8 (reviewed in Chen and Wang, 2012), and this primitive circulatory system is vital for ensuring the proper development of the embryo. Many different cell types coexist within the heart, such as muscle cells, vascular cells and pacemaker cells (reviewed in Chien et al., 2008). These different types of cell arise from a common pool of progenitor cells, but the details of this process and how these cells are maintained within the mature heart have puzzled cardiovascular scientists. Now, in *eLife*, Kathryn Ivey, Deepak Srivastava and co-workers at the Gladstone Institute of Cardiovascular Disease—including Amy Heidersbach as first author—have offered some important clues about the underlying mechanisms (Heidersbach et al., 2013).

The development of the heart is regulated at both the transcriptional and post-transcriptional level; regulation at the post-transcriptional level involves small non-coding RNA molecules called microRNAs (miRs). These molecules, which are evolutionarily conserved, regulate gene expression by binding to specific sequences within the messenger RNAs and reducing their stability or preventing them from being translated into proteins. Precise miR activity is required for the heart to develop normally, and for it to be able to respond to challenges such as insufficient blood supply, and pressure overload (the increased stress that develops in the left ventricular wall when the heart pumps blood)**.** Currently known cardiac enriched miRs include miR-1, 133, 206, 208 and 499 (reviewed in Chen and Wang, 2012), with miR-1 being the most abundant in the adult mouse heart.

MiR-1 is co-transcribed with miR-133a, and has two copies in the mouse genome, miR-1-1 on chromosome 2 and miR-1-2 on chromosome 18. Srivastava and co-workers have previously investigated the role of miR-1-2 during the development and maintenance of the heart (Zhao et al., 2007). In the current study, they have also involved its close relative, miR-1-1, in order to investigate the consequences of complete loss of miR-1 in the mammalian heart, and to identify any functions of miR-1 that are dependent on the amount of miR present.

Both miR-1-1 and miR-1-2 give rise to identical mature miR-1 species. Accordingly, targeted deletion of miR-1-1 results in a phenotype similar to that described for miR-1-2-null mice. However, the genetic background of the knock-out mice seems to affect the phenotype, as indicated by results from Heidersbach et al. and also those of another group who observed no phenotypic changes in miR-1-133 knock-out mice (Wystub et al., 2013). Nevertheless, both groups show that mice lacking all miR-1 copies (miR-1 null) die before weaning due to developmental defects. Heidersbach et al. observed abnormalities in mitochondria (the cell’s energy-producing organelles) as well as dysfunctional sarcomeres—the basic functional units of muscle fibres, which consist of thick filaments formed from the protein myosin and thin filaments made up of the protein actin.

Heidersbach et al. identified a gene called myosin light chain kinase (*mlck*) as being a direct target of miR-1. The *mlck* gene encodes an enzyme that adds phosphate groups to myosin molecules, and levels of this enzyme were increased in miR-1 null mice. However, this increased level of *mlck* was accompanied by a reduction, rather than an increase, in the phosphorylation of its substrate, the myosin light chain. This apparent contradiction was resolved by the discovery that miR-1 deletion leads to increased expression of an alternative form of MLCK, known as telokin, which lacks catalytic activity. Telokin is normally found only in smooth muscle—the type of weakly contractile muscle that lines the gut and the blood vessels—and its increased expression prevented MLCK kinase from phosphorylating myosin.

However, the fact that *telokin* alone was primarily induced—and not other forms of *mlck*, which all have regulatory sequences that are recognized by miR-1—implies that this might not be the whole story. Likewise, the dramatic increase in *mlck* expression (roughly fourfold) also suggests involvement of a more complex regulatory mechanism, since miRs generally change the expression level of their target messenger RNA by only 20–50% (reviewed in van Rooij and Olson, 2012).

When Heidersbach et al. analyzed the genes that were up-regulated in the hearts of miR-1 null mice, they found that levels of a protein called smMYOCD were dramatically increased. This protein, known as ‘smooth muscle version of Myocardin’, interacts with a transcription factor called SRF, which regulates the expression of genes that determine the characteristics of cardiac and smooth muscle. It turns out that smMYOCD is a direct target of miR-1. Moreover, the smMYOCD-SRF complex is more effective at promoting transcription of *telokin* than the cardiac version of Myocardin is. This completes a negative feedback loop that ensures that heart cells have a cardiac rather than smooth muscle phenotype: miR-1 suppresses smMYOCD, promoting the expression of the cardiac enzyme *mlck*, rather than smooth muscle genes such as *telokin*, resulting in phosphorylation of myosin and the formation of sarcomeres (Figure 1).

**Figure 1.**
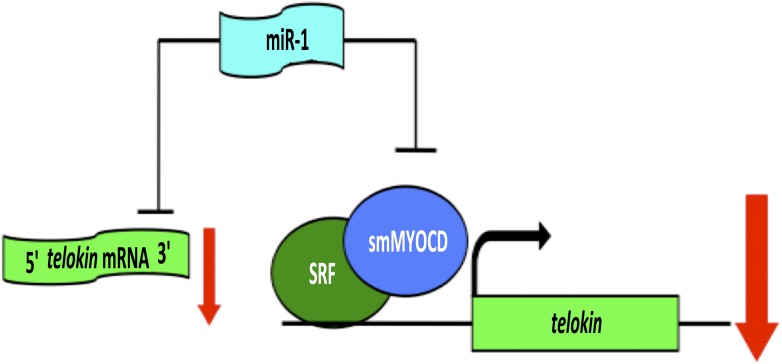
A network of genes regulated by a microRNA controls muscle development within the heart. The formation of highly contractile cardiac muscle, as opposed to weakly contractile smooth muscle, depends on the inhibition of a protein called telokin by a microRNA called miR-1. In addition to directly inhibiting the expression of *telokin* (left), miR-1 also inhibits the expression of *telokin* indirectly by reducing the expression of a protein called smMYOCD that, working with a transcription factor called SRF, activates the transcription of *telokin* (right). This combination of two different regulatory mechanisms guarantees that *telokin* will be inhibited in heart cells, ensuring the formation of highly contractile cardiac muscle.

This impressive feat of molecular sleuthing by Heidersbach et al. raises a number of important points that go beyond this specific story. Theoretically, miRs can target hundreds of genes across the genome due to the short target sequences (6–8 nucleotides) they recognize. The working model built by Heidersbach et al. illustrates how networks of genes regulated by miRs can indirectly control the expression of a critical gene in any tissue (Figure 1). Direct miR-mediated repression of a transcriptional activator for a target gene amplifies the influence of that miR on its target. In the example described by Heidersbach et al., the feedback loop comprised of miR-1, SRF and smMYOCD guarantees the sensitive and powerful regulation of *telokin* in response to environmental and pathological stimuli, and probably makes the heart more robust.

Heidersbach et al. also reveal the importance of computational methods to unwind the complex web woven by miRs in organ development and homeostasis. Future progress in this area will require creative computational approaches to help discover new mechanisms and principles of miR-regulated gene expression.
